# Milk Sickness: Its Etiology, Pathology, Diagnosis and Treatment

**Published:** 1859-03

**Authors:** F. R. Waggoner


*Milk Sickness: Its Etiology, Pathology, Diagnosis, and Treatment.*
By F. R. WAGGONER, M. D.

The subject of this article being one, as I believe, but imperfectly understood by the profession generally, and it having been my good fortune during the past few months to witness and treat a number of well-defined cases in my country practice, not knowing of a case except in the rural regions, I beg space in the *Peninsular and Independent*, to give to its readers and the Medical Fraternity the benefits of my observations and experience on a subject, to me, of great interest, and I think of vital importance to the Profession, hoping that it may prove of worth to some young Esculapian brother in the management of a fearful and often, in the hands of some, a fatal malady.

*Etiology.*—Its cause or origin is a subject of much speculation.—Medical men are as varied and differing in their opinions and conclusions as the non-professional and common observers. But, from the many hypotheses of its origin, but two, viz, the vegetable and telluric, carry with them any degree of plausibility.

The former of these is vindicated by a respectable and enlightened portion of the Profession. They say, that a certain species of vegetable, it not being known, abounds in the wood-land, and is matured by the latter months of summer, or first autumnal, at which season of the year the grass of the prairies becomes dry and tough, when the cattle resort to the timber for sustenance, feeding upon it, and, as the cow brute is very susceptible to its toxical influence, often sicken and die, while others, perhaps eating a less quantity, pass the season without ever showing signs of being poisoned by it. From such, careless and unsuspecting persons, using from day to day the milk, butter, and flesh of these animals, often fall victims to the disease.

Other observers, equally entitled to credence, contend that it is, as I intimated, of a *telluric* origin. Rising from the earth in the form of a vapor; or the nocturnal vapors, being conducting mediums, depositing during the night on the herbage, then communicated as in the former cases.

I repeat these are hypotheses that are warmly discussed by men of extended observation and research. But so far as my knowledge extends, I am forced to support the latter. Many facts can be adduced in support of its earthy, or telluric, origin, but a few will suffice for the present.

Where stock cattle, for instance are kept pent up until after the morning's dew, are never affected, though they are pastured where it is known to abound. Again, if feed, in the form of bundles of hay or fodder or sheaves of oats, have been cast on the surface of the earth where it was suspected to exist, fed to calves, or a calf, during the morning, while wet with dew, the result being the death of the animal.

Facts like these are, to my mind, evidence conclusive of its origin in the form of vapor. But let it originate from whence it may, it is only known in timbered land, and there disappears, after once cleared, cultivated, and seeded with tame grass; which shows, again, if of a telluric source, the toxical agent lies near the surface, and is destroyed by being shifted from its lurking place.

The length of this communication already admonishes me to say no more on its etiology.

*Pathology.*—In regard to this point I can say but little upon my own responsibility, as I have never made an analytic examination of the fluids; neither have I been permitted to make any *post-mortem* examinations of the vital organs. But judging from a clinical view, and from various manifestations, I conclude the *materies morbi* first enters the circulation, disorganizes the blood, which in turn passing through the nervous centres, producing prostration, languor, and other nervous pneumonia, not far removed from those witnessed in the Typhoid grades of fever. According to some observers, after death the spleen is found dark and congested, the brain softened, and the blood uncoagulated; showing a deficiency of plastic or coagulable lymph, though much, in my humble opinion, is yet vailed from the eye of the pathrologist.

*Diagnosis.*—No one that has ever once seen a case of this disease can fail in making a correct diagnosis. The physician is scarce ever called upon until the patient is in the second, or acute stage, of disease.

He generally finds the unfortunate sufferer with high gastric irritation, nausea, and vomiting. The stomach, no doubt, is much congested. The bowels are *uniformly*, *obstinately* constipated, and very tender upon pressure, sometimes swollen and pulsating, Prof. DUNGLISON to the contrary, notwithstanding. In his "Medical Dictionary," under the term "Milk Sickness," he writes thus:

"The symptoms of the disease are such as are produced by the acro-narcotic class of poisons—vomiting, *purging*, and nervous agitation."

No thing can be farther from the truth than there being *purging* in any stage of the disease. In no case has it occurred, that has fallen under my observation, nor the observation of other professional men in localities where it abounds of extended knowledge in the disease, from personal observation. I thus boldly speak that others, unexperienced, may not be misled by such statements.

*Tinnitus Aurium*, languor, and prostration are nearly always present. The pulse is generally accellerated, but weak; tongue much swollen and unwieldly, generally slightly coated with a dark brown fur; face flushed; eyes swollen much congested and red. The breath has a very peculiar fœtor; it is pathognomonic–an unmistakable evidence to the experienced olfactories. Extremities generally cold and clammy. A burning thirst, and constant desire for water

Such is a synopsis of the most striking diagnostic symptoms.

*Treatment.*—There are three prominent indications to fulfill: 1. Palliate the gastric irritabillity, allay vomiting and nausea; 2. Evacuate the bowels; 3. Support the patient.

The first of these indications is best fulfilled by frequent and full doses of the sulphate of morphine, together with the use of soda powders, etc. Also counter-irritation over the præcordium is of incalculable value in allaying the vomiting. An evacuation of the bowels is most easily attained by the use of mild and unirritating cathartics.—Oleum ricini, pulv. rhei, and the saline purgatives—sulph. magnesia, sodæ et potassæ, tartras, are the most potent agents. Drastic and irritating cathartics shonld be scrupulously avoided, together with the mercurials.

A few years ago the latter of these agents, mercury, was used to a great extent, but always attended I believe with bad results.

After thus succeeding in filling the two first indications we should have recourse to tonics and stimulants. The sulph. quinia in large, *very large*, and repeated, doses is, without doubt, the *sine qua non* at this stage of the treatment—though many other tonics are not without their beneficial effects.

Stimulants are of great importance. The alcholic are much preferable to all others—and the amount that can be tolerated is truly alarming. A patient I visited a few weeks ago had taken to the amount of a half gallon of whiskey within the past forty-eight hours, without any intoxicating or appreciable effects. The patient was a lady of medium size and stature—of nervous temperament. The vomiting had ceased, and the bowels had been evacuated by the use of some domestie agent. I at once had recourse to quinine. ℞. Sulph. Quinia 35 gr.; Sulph. Morphia ½ gr. M. Divided in seven equal portions; one taken every three hours. She convalesced within thirty-six hours. I also continued the whiskey, but very moderately.

I speak of no fatal cases, as my practice has been uniformly successful.—*Detroit Peninsular & Independent Med. Journal.*

